# Development, validation, and application of a South African Dietary Polyphenol Data Quality Assessment Tool

**DOI:** 10.1016/j.heliyon.2024.e40231

**Published:** 2024-11-07

**Authors:** Malory R. Jumat, Kwaku G. Duodu, Averalda E. van Graan

**Affiliations:** a Biostatistics Research Unit, South African Food Data System (SAFOODS) Division, South African Medical Research Council, Francie van Zijl Drive, Parow Valley, PO Box 19070, Tygerberg, 7505, Cape Town, South Africa; b Department of Consumer and Food Sciences, University of Pretoria, Private Bag X20, Hatfield, 0028, South Africa; c Department of Global Health, Division of Human Nutrition, Faculty of Medicine and Health Sciences, Stellenbosch University, PO Box 19063, Francie van Zijl Drive, Tygerberg, 7505, South Africa

## Abstract

There is a wealth of South African scientific literature studying and reporting on dietary polyphenols in research articles and academic literature. The evaluation of data quality is critical to ensure polyphenol data compiled from various scientific journals and academic texts are acceptable in terms of compilation standards. This study aimed to develop, validate, and apply a country specific South African dietary polyphenol data quality assessment tool (SADPDQAT). The evaluation tool considered nine evaluation categories with a maximum score of 5. For each literature source assessed, a quality score and confidence code was assigned. Validation analysis revealed generally consistent results between raters with an average percentage agreement of 65 %, no scores falling outside of Bland-Altman limits of agreement and a moderate internal consistency α = 0.533 which was improved to 0.616. Applying the tool to 383 scientific studies showed that most literature obtained a quality score between 70 and 79 % resulting in a confidence code of “B” (n = 166, 43 %) and a score between 60 and 69 % resulting in a confidence code of “C” (n = 125, 33 %). The SADPDQAT can be used to evaluate a variety of dietary polyphenol containing literature and aids in setting priorities for further research in this area.

## Highlights

• Research interest in dietary polyphenols has increased substantially.
• There are numerous South African scientific studies reporting on dietary polyphenols.
• The evaluation of data quality is critical to ensure that good quality polyphenol data is compiled.
• This study involves the development, validation, and application of a SADPDQAT.
• The SADPDQAT can be used to evaluate various dietary polyphenol containing studies.

## 1 Introduction

The positive association between plant food consumption and disease reduction has generated interest in identifying specific dietary bioactive compounds responsible for the observed health benefits. Polyphenols are secondary plant metabolites that comprise a heterogeneous group of molecules that constitute a large family of different compounds with highly diverse structures [1]. Polyphenols are common in many plant foods such as fruits, vegetables, cereal grains and beverages such as wine, beer and tea [2], which are food items frequently consumed by many South Africans [3], while the most common polyphenols in the human diet are flavonoids, phenolic acids, stilbenes and tannins [1]. Polyphenols consumption has been associated with multiple health benefits such as being cardioprotective [4,5], antidiabetic [5,6], anticancer [7,8], have possible antimalarial potential [9], promote gut health [10] as well as providing protection against neurodegenerative diseases [5,11]. Due to the variety of health benefits attributed to consumption of foods high in polyphenols, research interest in food polyphenols has increased substantially, especially among food scientists, nutritionists, the agricultural/food industry, as well as consumers [2,[12], [13], [14]]. Interest in dietary polyphenol research is gaining momentum as scientists endeavour to determine how their dietary consumption influences health. One example of their importance in recent health research is their role in the Dietary Inflammatory Index (DII). The DII is a system developed to assess the inflammatory potential of a diet as dietary polyphenol intake has been linked to a reduction in inflammation known to contribute to the development of multiple chronic diseases [15,16]. However, a key step in relating any health attribute to polyphenol consumption is the estimation of dietary polyphenol intake using a dietary polyphenol composition database.

**Fig. 1:**
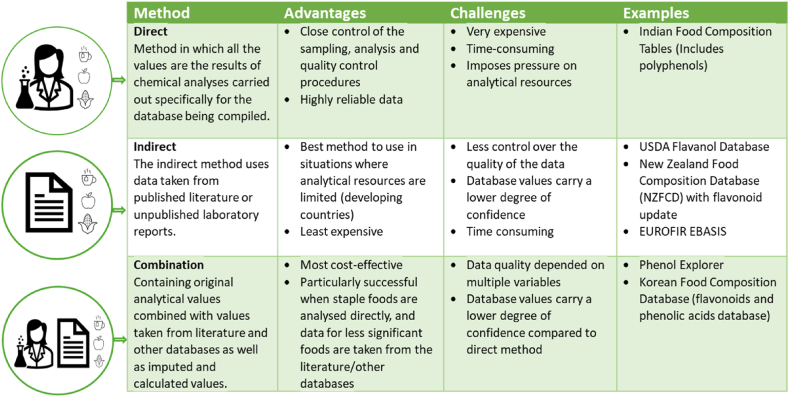
Methods used to compile food composition databases, their advantages, challenges and examples of polyphenol databases currently in use.

Food composition tables are broadly compiled by means of two methods, namely the direct and indirect method. Fig. 1 provides a summary of the advantages, challenges and current examples of the approaches used to compile polyphenol containing food composition databases. The direct method is considered the “gold standard” in food composition as all the values generated are the results of analyses carried out specifically for the database being compiled, and yields highly reliable data [17,18]. However, this is the most expensive route for food composition database development and is especially impractical for developing countries with limited funding, technical infrastructure and professional expertise [19,20]. Furthermore, methods for bioactives such as polyphenols are not yet as standardised as methods for nutrient analyses and proficiency testing schemes are not available [21]. Due to the vast and variable methodology available to analyse polyphenols in foods, to compile such a database solely using the direct method would not be feasible.

The indirect method uses data taken from published literature or unpublished laboratory reports. There can be multiple research reasons for determining a value for dietary component data which is not necessary performed with the aim of adding the value to a food composition database. For example, polyphenol data generated with the aim of optimising a methodological aspect of polyphenol quantification might not adequately report in sampling methodology, whilst studying the effect of environmental conditions on the quantification of a polyphenol will report sampling procedures in more detail but may have limited detail on the methodological aspect. Thus, data sourced through indirect methodology will more likely not adhere to the standard guidelines and not be optimised for food data compilation. Therefore, these data must be carefully considered and evaluated prior to their inclusion in the database [17,22].

The main international polyphenol databases currently used in polyphenol research are the European Food Information Resource Bioactive Substances in food database (EuroFIR-eBASIS) [23], Phenol-Explorer [24] and the USDA Database for the Flavonoid Content of Foods [25]. These databases were developed mainly using indirect or combination compilation methods as they contain polyphenol data extracted from primary research in the forms of scientific publications and peer reviewed thesis and dissertations. An example of the indirect method is the USDA's flavonoid database and EuroFIR-eBASIS. Whilst the Phenol-Explorer database was compiled with a combination of indirect and direct methods (Fig. 1). There are various examples of polyphenol compounds and classes contained in these databases and each database applies a unique quality evaluation system to extract data from peer reviewed scientific publications, highlighting the importance of quality evaluation of polyphenol data prior to publication in a database.

It is crucial to recognise that reliable compositional data and an accessible, comprehensive database that accurately represents the range of foods consumed within a country are fundamental in almost all quantitative nutrition research, dietary assessments, nutrition interventions and the creation of food and nutrition policies [26]. Failure to ensure polyphenol data quality prior to publication in a database could result in inaccurate study results and misleading conclusions leading to lack of credibility and confidence in the database. Furthermore, polyphenol data of poor quality could hamper data comparison and interchange [27]. In a recent systematic review Jumat et al. [28] found a wealth of South African scientific literature studying and reporting on a wide variety of dietary polyphenol data in research articles, as well as student dissertations and theses. This availability provides sufficient data to establish a dietary polyphenol database for South Africa. However, since it comprises indirect data, the evaluation of data quality becomes critical to ensure the polyphenol data compiled from the various scientific journals and academic texts are acceptable in terms of compilation standards [17].

As mentioned, polyphenols are a particularly diverse group of compounds and although there have been significant advances in methodology for their analysis [13,29], there are significant differences in the way these techniques are applied. Furthermore, the academic text and articles containing dietary polyphenol data were not typically done with database compilation as the focus. Therefore, food data systems recognized the need for data evaluation tools. The USDA has developed and endeavored to validate a multi-nutrient data quality evaluation system (USDA-DQES) [30]. However, this system is rather complex, was mainly developed for the North American landscape and literature and some aspects deemed important to polyphenol data were not individually assessed. This system is also specifically focused on flavonoid data and does not include other important polyphenols found in the human diet [2]. The EuroFIR eBASIS Critical Evaluation Scoring System (CESS) and guidelines for quality assurance were similarly not specifically developed to assess diverse polyphenol data [31], while the quality assessment method used by Phenol-Explorer does not focus on assigning data quality scores to scientific data [24]. Despite each of these data evaluation tools having aspects that are applicable for the evaluation of South African polyphenol data, they do not account for various aspects of polyphenol data deemed important in the South African context. For example, the tool should be easily interpretable, not require specialized equipment, be able to assess polyphenol data from a wide variety of academic literature, be applicable to a range of analytical methods as well as assess the critical aspect of polyphenol extraction separately. Additionally, the tool should be able to assign an indication of quality at the study level. Consequently, these individual data quality assessment tools were not deemed applicable to interchangeably assess South African academic texts that contain polyphenol data. Therefore, a tool structured to assess the diverse landscape of dietary polyphenol data found in South Africa was deemed necessary.

The aims of this study were thus to 1) develop a South African dietary polyphenol data quality assessment tool (SADPDQAT) specifically aimed at evaluation of South African dietary polyphenol data at an academic text level, 2) validate the data quality assessment tool for face validity and interrater reliability by applying the developed tool to a subset of polyphenol data containing literature and 3) to apply the tool to all articles systematically included to inform the development of a South African specific dietary polyphenol database to obtain an overview of the data quality and inform the priorities for data compilation and future analysis.

## 2 Methodology

**Fig. 2:**
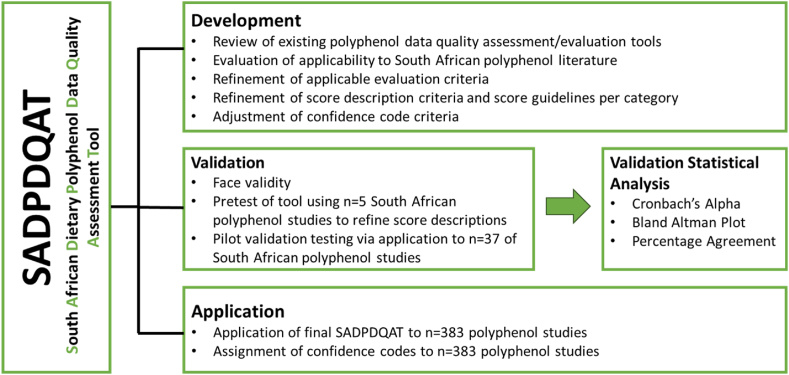
Flow diagram of the development, validation, and application of the South African Dietary Polyphenol Database Quality Assessment Tool (SADPDQAT).

The methodology applied for the SADPDQAT is described in three stages namely 1) development, 2) validation and 3) application, which is schematically presented in Fig. 2.

### 2.1 Tool development

The development of the SADPDQAT was based on the evaluation tools or systems of the three main polyphenol databases namely, the USDA multi-nutrient DQES, EUROFIR'eBASIS CESS as well as the Phenol Explorer's critical evaluation of data scheme. The EuroFIR system for evaluation of quality of data from scientific publications include six main criteria namely food description, sampling plan, sample handling, component description, analytical method and analytical performance [21]. The quality of a study was evaluated by providing a Yes or No response or assigning a score from 1 to 5 to each criterion which automatically calculates a critical evaluation score to each individual component. The USDA-DQES for flavonoids [22] was based on the procedures for evaluating multi-nutrient data [32]. All data in each study were evaluated for five quality categories namely number of samples, sampling plan, sample handling, analytical method and analytical quality control. The scores generated based on critical questions for each category were summed and converted to a percentage to provide a Quality Index (QI) from which a confidence code (CC) was assigned to individual data points in each study. Phenol Explorer subjects articles containing quantitative data on polyphenols to a less structured evaluation system based on sample, analytical method and expression of results [24]. Data were not assigned a specific quality score but their inclusion in the database was based on meeting several minimum requirements. A summary of the advantages, limitations and overall applicability to be used in a SADPDQAT is presented in Table 1.

**Table 1:** Overview of the main advantages, limitations and applicability of the data quality evaluation systems for the three major polyphenol composition databases.

	Advantages	Limitations	Applicability
USDA Data Quality Evaluation System (DQES)	Easy to interpret Included confidence codes	Flavonoids only HPLC methodology only	Good
EUROFIR eBASIS Critical Evaluation Scoring System (CESS)	Comprehensive and detailed	Complicated – designed with more advanced countries in mind Considers only advanced analytical methods Reviewers require special training	Limited
PHENOL EXPLORER's Critical Evaluation of Data	Easy to interpret Considers multiple analytical methodologies	Not too comprehensive – no clear document/scheme No indication of quality to user (no scores)	Good

eBASIS - Bioactive Substances in Food Information; HPLC – High Performance Liquid Chromatography.

The three main polyphenol data evaluation systems [21,22,24,32] were critically analysed for their applicability to evaluate South African dietary polyphenol literature. Each system's individual evaluation categories were scored a point from 1 to 3 based on their applicability to South African polyphenol literature. A score of 1 indicated that the specific category is not applicable and would thus not be used in the tool development. A score of 2 indicated relative applicability and thus the category would need further exploration. A score of 3 indicated high applicability and thus the category would be included in the development of the South African Dietary Polyphenol Literature evaluation tool. Furthermore, chapters 8 to 10 of Food Composition Data: Production, Management and Use [17] was consulted to provide detail to the tool categories and score descriptions as well as assist with the appropriate language in the score guideline section of the tool.

### 2.2 Tool validation

The SADPDQAT was tested for validity and reliability in three parts: (I) face validity, (II) pre-test and pilot, (III) literature assessment and validation.

Face validity indicates whether the measurement categories are relevant and measure what the tool claims to measure [33]. Face validity of the SADPDQAT was performed by two academic professionals with advanced experience in the field. Evaluators were asked whether the categories were applicable for the evaluation of South African polyphenol literature for data composition and whether the categories, scores per category, score descriptions and guidelines were clear.

The pretest sample consisted of five relevant randomly selected academic texts and was evaluated independently by evaluators who have completed the FAO/INFOODS Food Composition training as well as the Global Challenges Research Fund Workshop on Production, Management and Use of Food Composition Data. Furthermore, evaluators had extensive experience on the production, management, and use of food composition data. The objective of the pretest was to ascertain whether the instrument necessitated additional modifications after meticulously reviewing the randomly selected polyphenol literature, identifying aspects within the rating categories that needs further refinement to improve clarity and inter-rater reliability. For example, if evaluators’ scores differed by three or more points on a particular category per literature record, the relevant session was discussed in detail, modifications were made, and the section was re-evaluated by both evaluators until agreement was reached.

Once consensus was reached and the evaluation criteria, score indicators and guidelines were set, the pilot validation study was conducted by both evaluators by applying the SADPDQAT to a random selection of 10 % (n = 38) of polyphenol studies that were systematically included to inform the development of the South African Dietary Polyphenol Database [28]. One record was excluded as it was found to not contain quantitative polyphenol data, while the 37 records used comprised of 18 scientific articles and 19 dissertations/theses. The evaluators independently scored the studies using the 9-category SADPDQAT. The completed evaluation forms were captured in Excel sheets and statistically analysed to assess the response for each record.

Statistical analysis using Cronbach Alpha coefficients indicated that removal of the evaluation category - number of primary samples, would improve the tools internal consistency to α = 0.616. To improve the tools internal consistency reliability, the category concerning the number of primary sample category was removed. A relatively weaker coefficient was also observed with the sampling method category. Recognizing the significance of sampling plans in food composition [17], the category was rather modified to simplify score criteria and enhance clarity. The tool was thus adapted accordingly, and the final 8-category data quality assessment tool was applied in the assessment of the 383 studies (one study was excluded as explained above) systematically sourced to inform the development of the dietary polyphenol database.

### 2.3 Tool application

The final 8-category data quality assessment tool was applied to 383 included studies identified by Jumat et al. [28]. Each study was awarded a confidence code based on the percentage obtained when the scores for each category was summed and converted to a percentage. The confidence codes detailed for this evaluation (Table 3) helps to identify studies that are eligible for compilation in the reference dietary polyphenol database and ultimately in the user database. Furthermore, the assigned confidence codes assist in identifying and setting priorities for foods that need to be chemically analysed while also orientating researchers to generate high quality dietary polyphenol data [34].

### 2.4 Statistical analysis

The statistical software for data science (Stata), version 18 was used for statistical analysis. The internal consistency of the SADPDQAT scores was calculated using Cronbach's alpha. This indicates the degree to which items measuring the same general construct produce similar scores [35]. Agreement between the raters were analysed using Bland-Altman plots [36], whilst inter-rater reliability was determined with percentage agreement analysis [37]. Mean, minimum and maximum scores per category and their standard deviations per evaluation category of the SADPDQAT were calculated using Microsoft Excel version 16 (Microsoft, 2024).

## 3 Results and discussion

### 3.1 Tool development

In total, 15 data quality categories were identified across the three food composition data quality assessment tools and relevant literature, while 10 were deemed highly applicable to South African polyphenol literature. Upon further review, one category was found to be repetitive and was removed. The final list of categories deemed to be highly applicable were 1) Food Description/Identification, 2) Number of Primary Samples, 3) Sampling Plan, 4) Sample Handling and Processing, 5) Analytical Method, 6) Number of Analysed Samples, 7) Extraction Method, 8) Component Identification/Expression of Results and 9) Analytical Quality Control.

It was important to develop a tool that is relatively easy to interpret, did not require specialized equipment and can be applied across the very wide variety of South African polyphenol composition data. Therefore, a description of each category and the reason for addition to the SADPDQAT is detailed in Table 2. Each category had a maximum score of 5 when all the requirements for the category was fulfilled. A score of 0 was awarded when no information for the category could be found. This range of scores allowed evaluators to account for nuances in the South African polyphenol literature and thus provide more representative scores. For each score a short description for the point was indicated to warrant awarding of the score. Reviewers also had access to a detailed scoring guideline per category to ensure that reviewers understand the objective of the score and how to apply it to polyphenol literature.

**Table 2:** An overview of the SADPDQAT data evaluation category descriptions and scoring criteria guidelines.

Category	Description and justification	Scoring criteria guidelines
Food Description/Identification	Identifies that the correct food is being evaluated and that the naming of the food is clear and unambiguous. There are many factors that can influence food nutrient content and these factors need to be reflected in the food description [21].	• A score of 5 was awarded if the food was described in terms of the scientific name, cultivar, and/or breed, part of plant analysed, state of maturity, food form (fresh, frozen, dried, canned, cooked, prepared, preserved, etc.), traditional/vernacular name, type or brand if processed, with an indication of food measurement. • A lower score was awarded when the above mentioned were not apparent in the publication.
Number of Primary Samples	Refers to the units collected from the catchment area/total population of the food and is the starting point in compositional studies [17]. This category indicates that an adequate number of samples were collected to provide a good estimate of the polyphenol value.	• To score 5 > 30 samples had to be collected. • Lower scores were awarded for fewer number of samples. • If there was a polyphenol value but no information found in the document, number of primary samples were assumed one, and awarded at least 1 point.
Sampling Plan	This category was added to reflect the representativeness of the food sample from which polyphenols were analysed. Holden et al. [32] found that many published reports use convenience sampling and experimental farms leading to a loss of representativeness.	• 5 was awarded for a national sampling plan with supporting statistics across multiple provinces/geographical areas and seasonality [17]. • Scores of 3 and 4 were awarded if foods were sampled from multiple regions representative of the food analysed [38]. Convenience sampling and experimental farms were included at the lower end of the scale.
Sample Handling and Processing	Polyphenols are susceptible to environmental exposure and incorrect sample handling and processing will inevitably yield incorrect content values. The stability of polyphenols in food matrices is influenced by pH value, light, temperature, oxygen, metal ions, enzymes, proteins, nitrite salt, sulfur dioxide, other antioxidants, and interactions with other food constituents [39].	• A score of 5 was only awarded if analyses was on the edible portion, there was an explanation of how the analytical sample was obtained from the primary sample, details were provided on sample preparation, storage, storage state, temperature, storage container and moisture content was provided. • Progressive deviations from these were awarded a concurrent lower score.
Analytical Method	Evaluation of the *Analytical metho*d is especially important in polyphenol analysis as there currently is still no gold standard or universally accepted method for their analysis [40,41] therefore this category was not mainly scored on the type of method used but rather on the adequacy and detail in which the method was documented.	• 5 was only awarded if the analysis was performed by an accredited laboratory, clearly stated in the text. • A score of 4 was awarded if analytical method was explained in detail including the use of appropriate standards and reference materials. • Lower scores are indicative of a less detailed analytical method description.
Number of Analysed Samples	*Number of analysed samples* gives an indication of the mean and generally, the more samples the better estimation of the mean. Nutrient analysis done in triplicate is usually the acceptable standard for chemical analysis as primary samples are often combined to form composites to reduce costs of analysis [17].	• The maximum score of 5 was awarded when more than 5 replicates were analysed. • Lower scores coincided with a lower number of replications.
Extraction method	Due to the vast chemical diversity of dietary polyphenols, extraction of the polyphenols from the food matrix is an important step in quantifying phenolic compounds [40]. There are also several food and polyphenol-specific techniques for extraction thus the scoring of extraction method was determined by how detailed the methodology was documented.	• A score of 5 was awarded if the extraction method listed the solvent type and concentration, the apparatus and materials used, as well as the temperature at which extraction took place. • A bonus point was scored if the rate of extraction was listed. • If foods were analysed directly (no extraction was needed due to the nature of the sample and analytical method e.g. wine or juice analysed using chromatography) a maximum score was awarded.
Component identification/expression of results	Food composition data is generally reported per 100 g of edible portion [17] and the component identification/expression of results category was thus deemed critical as this indicates to the compiler that the value needs recalculation as it is expressed in a manner different to the database.	• Results reported per 100 g of edible portion received the maximum score. If the value could easily be calculated with unit conversions a score of 4 was awarded. • Results reported in analytical format needs more sophisticated calculations that can result in loss of analytical quality control indicators and thus a maximum score of 3 was awarded. • Values expressed in graphs but also found in text were awarded a score of 2.
Analytical quality control	Analytical quality control provides an indication of the magnitude of variability of the data and reflects the performance of the method with respect to accuracy, and the standard deviation reflects its precision.	• A low relative standard deviation (≥5 %) indicate that the method used precision is high and is thus awarded a maximum score. • Scores decrease with an increasing relative standard deviation. If standard deviation was not detailed a maximum score of 1 was awarded.

**Table 3:** Confidence codes assigned to assessed South African dietary polyphenol data.

Confidence code	Percentage	Interpretation
A	80–100	The literature assessed is of very high quality and user can use polyphenol values with considerable confidence.
B	70–79	The literature assessed is of high quality and user can use polyphenol values with significant confidence.
C	60–69	The literature assessed lack some quality aspects, but the user can still use the polyphenol values with confidence.
D	50–59	The literature assessed lack several quality aspects, the user should use the polyphenol values with caution.
None	<50	The literature lacks critical quality aspects and thus there is no confidence with using polyphenol data derived from this literature.

The total score out of 45 was converted to a percentage and from this percentage a confidence code was assigned. The confidence code assignment was modified from Holden et al. [32] to be more representative of the diverse nature of the South African polyphenol literature and is described in Table 3. Any literature that scored lower than 50 % overall would not be included in the database.

One of the key features of the SADPDQAT is the wider range of assessment categories, as it assesses 9 categories while the USDA-DQES and EUROFIR-eBASIS CESS assess 5 and 6, respectively. The wider range was intended to account for the variety of studies as well as the variety of polyphenols to be included in the database. Due to the importance of polyphenol extraction, the SADPDQAT added the extraction method criteria separately. The analytical methods category of the SADPDQAT was not as restrictive on the scientific method used to analyse the polyphenols such as the USDA-DQES which only evaluates data generated by HPLC methods. Whilst the quality evaluation applied by Phenol-Explorer does not provide quality scores/indices and thus users do not have the ability to gauge data quality directly from the database.

### 3.2 Tool validation

The academic experts did not suggest changing the category types or score indicators, indicating that the tool has face validity. Minor revisions resulted in modification to the wording and scoring guidelines in some categories to enhance the clarity and avoid ambiguity of the sections. Both reviewers however agreed that the 9-categories were adequate in assessment of dietary polyphenol data quality.

**Fig. 3:**
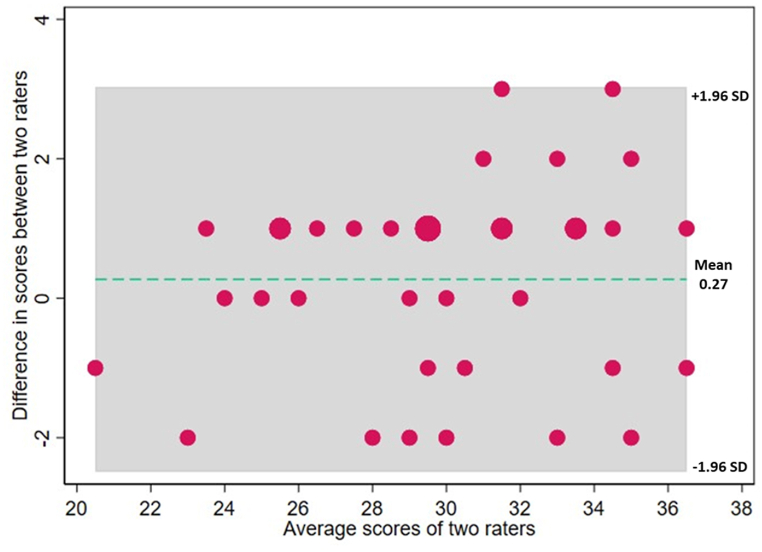
Bland-Altman plot representing the agreement between evaluators applying the SADPDQAT to n = 37 polyphenol studies during the validation phase.

Interrater reliability was measured using Bland Altman plots as well as percentage agreement analysis. Fig. 3 shows Bland Altman plots of agreement which shows the average difference plotted against the mean scores between the raters. The mean difference of 0.27 indicates a very low average difference in scores between the raters. The limits of agreement were between −2.5 and 3.0 and no scores fell outside of the limits of agreement. Average scores were between 20.5 and 36.5 indicating good agreement and therefore interrater validity between evaluators. Furthermore, the primarily random scattering of points indicates that the observed differences are not dependent on the magnitude of the measurements [36].

**Fig. 4:**
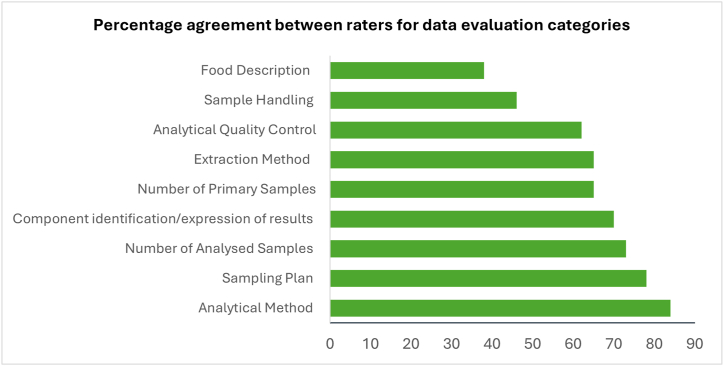
Percentage agreement amongst evaluators for the 9-category validation set of polyphenol studies (n = 37).

It has been observed that interrater reliabilities are affected by the fineness of discriminations in the data that collectors must make. If a variable has only two possible states, and the states are sharply differentiated, reliability is likely to be high, whilst reliability is difficult to obtain when observations with finer discriminations are evaluated [37]. Percentage agreement between the evaluators ranged from 38 to 84 for the nine evaluation categories across 37 polyphenol studies (Fig. 4), with an average of 65. The lower agreement percentages were observed for food description (38) and sample handling (46), while sampling plan (78) and number of analysed samples (84) scored high agreements. There was multiple “finer” discriminations within the food description and sample handling criteria possibly explaining the lower percentage agreements observed. However, the sampling plan and number of analysed samples categories possibly scored higher since their evaluation criteria were more specific.

When applying the SADPDQAT to the validation set (n = 37) of polyphenol studies, food description, analytical and extraction method evaluation criteria scored highest with relatively low variation (Table 4). Moderate scores were obtained for sample handling, number of analysed samples and component identification. Lower scores were observed for sampling plan, and analytical quality control. Food description, sample handling, analytical method and component identification indicated the smallest range of scores, while sampling plan and analytical quality control had the widest range of scores. It is thus clear that there was limited data on sampling plan across the validation set of data. It was observed that the polyphenol literature assessed for this study was often not designed for food compilation, and therefore the critical aspect of sampling was not deemed important and limited information was mentioned in the methodology sections of the studies. Those that did provide sampling data, mostly made use of convenience sampling. The analytical methods category scored high with a low variability, indicating that the studies all reported the analytical method used for polyphenol quantification in sufficient detail and the evaluation categories were specific enough that both reviewers scored this section concisely. The number of primary samples category also scored lower than average with large variance. From the validation set it was found that this data was either not described clearly, leading to assumptions needing to be made or this information was not reported at all. The intention behind creating a separate category for the evaluation of the number of primary samples from sample handling and number of analytical samples was to limit confusion amongst evaluators between the primary and analytical samples as well as prevent penalizing a study if this information was not reported but overall quality of the study is good, indicating that the value is useable in a first edition polyphenol composition database. During their validation study, Bhagwat et al. [30] also found that the number of samples category (which includes evaluation of primary samples), in the USDA-DQES was one of the most difficult categories to evaluate.

**Table 4:** Mean, minimum and maximum scores per category of validation set of data (n = 37) of evaluators using the 9-category SADPDQAT.

Evaluation Category	Mean (SD)	Min-Max
Food Description	4.2 (±0.7)	2–5
Number of Primary Samples	2.9 (±1.8)	0–5
Sampling Plan	1.4 (±0.9)	0–4
Sample Handling	3.6 (±0.7)	2–5
Analytical Method	4.0 (±0.3)	2–5
Number of Analysed Samples	3.5 (±1.4)	1–5
Extraction Method	4.0 (±0.8)	1–5
Component identification/expression of results	3.5 (±0.7)	2–5
Analytical Quality Control	2.8 (±1.8)	0–5

SD – Standard Deviation.

The analytical quality control had a low mean score and highest variability. It was observed that many of the studies did not report the percentage relative standard deviations in the data. If they did report on variability, it was only the standard deviation and thus to get to the % relative standard deviation required additional calculations and interpretation. Additionally multiple studies making use of chromatography for analysis, seemingly only analysed one sample as these studies did not report any variability in the values reported, resulting in a low score. Interestingly, Bhagwat et al. [30] also found that this category was the most difficult to evaluate as it requires understanding of the underlying concepts and it is also the most neglected category reported in publications.

Applying Cronbach's alpha to the scores assigned to the validation set of polyphenol literature to measure the overall internal consistency reliability of evaluators for the 9-category SADPDQAT found a moderate internal consistency (α = 0.533). Though none of the three main polyphenol database evaluation tools (Table 1) have data on the internal consistency of their tool or scheme, assessment of other nutritional tools similar in scope to the SADPDQAT have also observed lower Cronbach alpha scores. For example, a Cronbach's alpha value of 0.502 has been found in the development and validation of an assessment tool to assess online nutrition information [42]. Furthermore, reliability assessment for a dietary tool aimed at the assessment of diet quality in Indian adolescents also obtained an alpha of 0.50 [43]. Another study aimed to evaluate the reliability and validity of the well-known and widely used full and short form of the Mini Nutritional Assessment tool among the elderly in Ethiopia and observed an alpha of 0.5 for the short form [44]. The above mentioned thus suggest that reliability of some nutrition quality assessment tools is inherently low due to the heterogenous and wide range of the type of data being evaluated. However, statistical analysis did indicate that the removal of the *Number of Primary Samples* category increased the alpha value to 0.616, which is considered acceptable and indicative of a higher internal consistency. Therefore, the SADPDQAT was modified by excluding the *Number of Primary Samples* category prior to application of the tool.

To explore the tool's ability to assess polyphenol compilation data quality of similar foods across various research contexts and study objectives all literature containing tea (rooibos, honeybush and bush) from the validation data set was extracted and presented as a case study in Table 5. From the data presented it can be observed that a wide variety of study objectives are evident whilst generating quantitative polyphenol data for tea. Data quality scores between raters were mostly closely related. However, raters differed on eight out of the nine quality assessment criteria with the assessment of the study where chemometric analysis and kinetic modeling was applied to study the phenolic changes of rooibos tea during fermentation. Furthermore, different scores between raters were observed for six out of the nine evaluation criteria for a study that aimed to develop a xanthone enriched honeybush tea extract and also employed advanced chemical techniques. Indicative that though they score overall high data quality scores that discrimination between score levels was challenging. A possible reason could be that the type of technology used in these studies are unfamiliar to the raters and interpretation of the various aspects may be subjective. Interestingly, only one study showed differences for scores of the analytical method, corresponding to the high percentage agreement for analytical method across the validation data set (Fig. 4). However, for eight of the assessed tea studies the raters differed in their scores for food description. This was interesting as foods were generally identified adequately in terms of their descriptions (Table 4), however, some quality aspects were unclear or “hidden” across the documentation as authors did not always specify which portion of the plant was used for analysis, the state of maturity or the sample size in the methodology sections of the papers, leaving room for assumptions based on information found elsewhere in the paper. Nevertheless, overall scores only differed with 1–2 points (data not shown), equating 2–4 %. Thus, quality scores between raters were still closely related, though more detailed score definitions or ranges might increase the tool's ability to discriminate the finer details of the diverse data set.

**Table 5:**
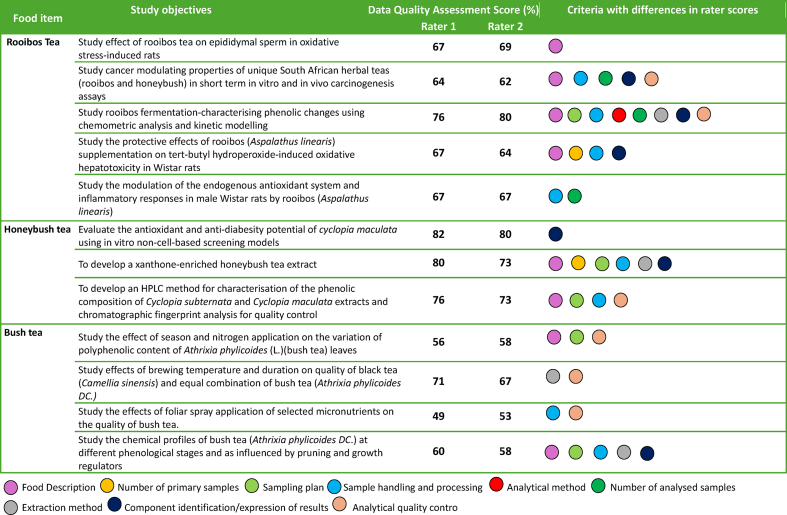
Tea case study presentation of rater score differences when assessing various tea food items from the validation data set.

### 3.3 Tool application

**Fig. 5:**
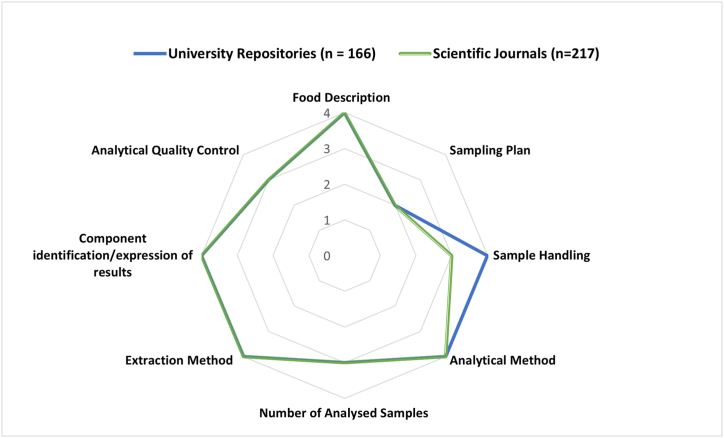
Radar chart of the data quality assessment scores assigned to data from university repositories and scientific journals.

Across the 383-polyphenol data-containing papers that were evaluated using the SADPDQAT foods were well described with a mean score of 4 for food descriptions (Fig. 5). The component identification/expression of results, extraction and analytical method categories also yielded good scores. Interestingly, sampling plan scored the lowest, similar to the scores assigned for this category in the validation sample (Table 4). This indicates that majority of the studies assessed either did not report this critical aspect in their methodology or made use of convenience sampling or experimental farms. Though convenience sampling does not generate high confidence in the values obtained [17], there are various reasons why analyst will make use of convenience samples. A major reason is the high costs of sampling and coupled analysis [45], the fact that many foods high in polyphenols are often only cultivated in one province/geographical region such as rooibos tea [14], and that the primary aim of the study was not to obtain representative polyphenol data for compilation. For example, a study evaluating the quantitative and qualitative loss assessment along the postharvest supply chain of lettuce [46] scored a 4 for sampling plan as they included lettuce from various commercial farms across two provinces. A study evaluating the phytochemicals and overall quality of leafy lettuce varieties grown in closed hydroponic systems [47] scored a 1 for sampling plan as they made use of experimental farms. Both studies analysed similar polyphenols in lettuce but with different objectives which inevitably affected their sampling procedure and average scores.

**Fig. 6:**
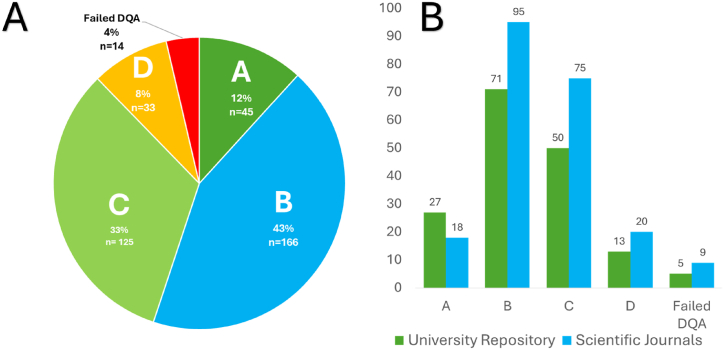
Distribution of confidence codes assigned to total amount (n = 383) of literature sources evaluated (A) to inform the development of a South African Dietary Polyphenol Database *and* university repositories and scientific journals (B). DQA – Data Quality Assessment.

Out of the assessed studies, 12 % (n = 45) obtained a data quality assessment score of 80 % or above (Fig. 6A), which corresponds to a confidence level of A (Table 3), while the 14 studies that failed the data quality assessment will not be assigned a confidence code and will thus be excluded from compilation in the database. Most studies obtained a confidence code B (43 %) and C (33 %) indicating that though not all evaluation categories scored high, the studies contain good quality polyphenol data, and the polyphenol values can be used with significant confidence. Similar trends were observed when the USDA-DQES was applied to the USDA's flavonoid database [22], with majority of studies sourced receiving confidence codes of B (61 %) and C (31 %). Modification and application of the USDA-DQES to Brazilian flavonoid data, resulted in most of the data being assigned a confidence code of C (99 %), which was attributed to the low scores obtained for the number of samples evaluation category [34]. The higher quality scores found when applying the SADPDQAT to the systematically sourced South-African polyphenol data can be due to the exclusion criteria already set out by Jumat et al. [28]. Thus, studies which did not conform to the inclusion criteria (non-duplicated data, values in tables or text, South African foods, polyphenols quantified, food item is typically consumed as food and analyses done on edible portion) and would have inevitably obtained a lower quality score were already excluded. The data that failed quality assessment consistently scored very low in three categories – the sampling plan, sample handling and analytical quality control similar to the results obtained for the validation data set (Table 4).

The university repositories (dissertation and theses) yielded more A confidence codes compared to scientific journals (Fig. 6 B). The reason for the higher scores can be attributed to the fact that dissertations/theses usually contain more detailed information for the various aspects of a study, compared to scientific articles often being limited to prescribed length restrictions, omitting detailed background information to favour results and discussions.

The SADPDQAT demonstrated applicability to a wide variety of dietary polyphenol data containing studies, using a variety of sampling approaches and analytical methodology. This applicability was strengthened with the application of statistical validation methods. Additionally, the overall quality of South African polyphenol data was good with 12 % of studies (n = 45) scoring a confidence code of A and 43 % (n = 166) of studies receiving a confidence code of B. A notable limitation of this study is based on the complex nature of polyphenol analysis. Analysing dietary polyphenol data is a complex task for database compilers as it requires in depth knowledge of a variety of food matrices and analytical terms, further complicated by the vast variety of polyphenol compounds and their analytical methodology [13]. Thus, raters applying any tool developed for polyphenol data assessment will require additional training to increase understanding of polyphenol analysis. Validity can be improved in future by including evaluators across a wider range of food compilation related fields such as food analysts, food scientists, food technologist, nutritionists, and dietitians and to further refine the assessment criteria within the different categories. This will show that the tool can be applied across various food compilation database users and not just generators. Even with scoring guidelines provided there were instances where assumptions had to be made. These assumptions could affect the validity of the tool and therefore needs to be limited.

Suggestions for future amendments of the tool were identified: due to the fact that the compilation of polyphenol composition is less common than that of nutrient compilation, some terms may be unfamiliar to evaluators who are experienced in general food compilation but not specifically in polyphenol analysis, which could make the evaluation of these works more difficult. Therefore, including an annexed glossary of terms commonly used in polyphenol analysis with the tool would enable evaluators to look up terms to avoid confusion. Another suggestion is to expand and refine the sampling plan scoring criteria. During the evaluation process, it was observed that a study's sampling plan would often have a better fit between two score categories. Therefore, expanding the assessment criteria/categories and score options could enhance the accuracy of scores assigned by evaluators.

## 4 Conclusions

The development, validation and application of the SADPDQAT concludes that generally consistent results can be obtained by raters evaluating a wide variety of South African dietary polyphenol containing literature. Furthermore, most South African polyphenol studies evaluated in this study can be confidently compiled in a dietary polyphenol database. To the best of our knowledge, this constitutes the first attempt at the development, validation and application of a tool geared to assess South African polyphenol literature with the aim of applying assigned confidence codes in the South African Dietary Polyphenol Database to guide database users and contributors on the data quality. The application of the SADPDQAT to South African polyphenol literature can be useful in identifying and prioritizing gaps that could inform further research.

## CRediT authorship contribution statement

**Malory R. Jumat:** Writing – original draft, Visualization, Methodology, Investigation, Formal analysis, Data curation, Conceptualization. **Kwaku G. Duodu:** Writing – review & editing, Supervision, Methodology. **Averalda E. van Graan:** Writing – review & editing, Supervision, Methodology, Investigation, Conceptualization.

## Disclosure

This work has not been published nor submitted to any other peer-review journal for publication. During the preparation of this work the authors used Chat GPT and QuillBot only to improve readability. After using these tools, the authors reviewed and edited the content as needed and takes full responsibility for the content of the publication.

## Data availability

Data will be made available on request.

## Declaration of competing interest

The authors declare that they have no known competing financial interests or personal relationships that could have appeared to influence the work reported in this paper.
